# Understanding Consumers’ Intentions to Purchase Clean Label Products: Evidence from Taiwan

**DOI:** 10.3390/nu14183684

**Published:** 2022-09-06

**Authors:** Min-Yen Chang, Han-Shen Chen

**Affiliations:** 1 Department of Accounting, Jiaxing University, Jiaxing 314001, China; 2 Department of Health Industry Technology Management, Chung Shan Medical University, Taichung 40201, Taiwan; 3 Department of Medical Management, Chung Shan Medical University Hospital, Taichung 40201, Taiwan

**Keywords:** food quality labeling, food safety, health consciousness, food marketing, consumer food choice behavior

## Abstract

In light of the fact that increasing consumer emphasis is being placed on the concepts of safety, health, and environmental protection, and that consumer groups are now attentive to the issues of “greenism” and sustainable development, the certification label has become an important tool. This study explores individual needs from the perspective of food “clean label” certification, highlighting that the importance of general food certification to consumers is different from the previous literature that only focused on the impact of organic labeling, nutrition labeling, and food safety certification on consumer behavior. In this study, the consumer purchase intention for the use of the “clean label” and its influencing factors are discussed, using product knowledge and involvement as the independent variables. The target is the consumer who has experience with “clean label” products. This study employs snowball sampling. A formal questionnaire was sent to 292 participants. After eliminating the invalid samples, we retained 265 valid questionnaires for the analysis (a valid response rate of 90.75%). Structural equation modeling (SEM) was applied to test the research hypotheses. The results indicated that: (1) consumers’ involvement with “clean label products significantly influences their purchase intention; and (2) consumers’ product knowledge of “clean label” products significantly influences their purchase intention and involvement. Based on these results, enhancing consumers’ knowledge of food security is suggested. Furthermore, the findings provide crucial insights for marketing channels, suggesting that the food industry can target consumer confidence over certification labeling and “clean label” products as keys to purchase intention, and to attract business by developing practical marketing strategies.

## 1. Introduction

In recent years, food contamination accidents have been frequently reported. For example, in 2016, an illegal factory in Tainan, Taiwan mixed edible pigments and industrial dyes to make tangyuan. In 2017, the chicken eggs at 44 farms in Taiwan were found with excessive Fipronil levels. Food contamination accidents have affected Europe in recent decades, such as those related to BSE and dioxin [1,2]. Consumers are concerned about the use of artificial ingredients, additives, and colorants such as E133 [3], and the adoption of controversial food technologies such as GMOs [4]. This concern has prompted consumers to become skeptical or worried about the adverse health effects of the food system [5]. In addition, many current food products are harmful to human health [6]. 

As the business opportunities for healthy food continue to expand, the phenomenon of mislabeling in the market has seriously affected the health and rights of consumers. It has also made many consumers doubtful about the claims made by the industry, and they require clear and reliable product information [7,8]. Consumers are increasingly concerned about healthy behaviors in their lifestyles and their eating habits [9,10]. They require foods that are natural and organic [11,12,13,14], less processed and “free” of ingredients that are negatively rated in various ways, such as allergen-related ingredients or additives [15,16].

Many food consumers appear concerned about these impacts of the food system, as is evident from the increasing demand for environmentally friendly food products [17]. As a result, consumers are willing to purchase organic products that are more respectful of human health and the environment [18,19]. However, messy and fuzzy food information may cause consumers to make incorrect decisions. Certain foods on the market flaunt the “clean label”, which emphasizes a simple process, the avoidance of unnecessary food additives, and palatability. However, consumers are still unfamiliar with the “clean label”, and different consumers have different perceptions and directions of interest, which raises interest in the study. 

Ingredion [20] recommends that a “clean label” product is one that is positioned as “organic”, “natural” (i.e., following natural production methods), and/or “free of” artificial ingredients/additives. Nonetheless, the “clean label” designation is most often attributed to foods based on the presence or absence of certain ingredients (e.g., additives and preservatives) used in the food [20,21]. There are multiple factors (e.g., environment, sustainability) involved in the “clean label movement” [22,23,24,25,26]. Food composition-related health issues [7,22,26] and trends in health and sustainability are driving consumers to consider which ingredients are used in the foods they consume in their daily lives [27]. However, messy and fuzzy food information may cause consumers to make incorrect decisions. Food manufacturers are addressing these trends by offering “clean label” foods. 

“Clean label” products are characterized by the presence of ingredients that are considered natural, harmless, and simple, and that consumers know and use as their own ingredients [20,28,29]. The food industry is meeting the growing consumer demand for such “clean label” products by offering foods that feel cleaner [30]. For example, in 2010, Heinz tomato ketchup was reformulated to remove the high fructose corn syrup from the ingredient list and renamed it Simply Heinz [30]. Certain foods on the market flaunt the “clean label”, which emphasizes a simple process, the avoidance of unnecessary food additives, and palatability. However, consumers are still unfamiliar with the “clean label”, and different consumers have different perceptions and direction of interest, which raises interest in the study.

In fact, many consumers think more about the “clean label” when buying food [31], rather than brand awareness. A survey by the Center for Food Integrity found that 75 percent of respondents identify nutrition and ingredient labels on their food; 53 percent believe that “clean label” products were healthier. Notably, 46% of Americans say that the availability of food ingredients expressed in a simple way directly affects their purchasing decisions, and that they are willing to pay more for these foods [32]. Therefore, the market size of “clean label” ingredients food is likely to grow substantially; it is expected to reach approximately USD 47.5 billion by 2023, registering a CAGR of 6.8% during the forecast period [33].

Most of the literature in the past focused on the impact of organic labeling, nutrition labeling, and food safety certification on consumer behavior [34,35,36,37,38,39,40,41,42]. Delgado-Pando et al. [43] explored potential “clean label” alternatives to the most common additives in meat products (e.g., antimicrobials, antioxidants, thickeners, and colors), as well as some new technologies for developing clean-label meat products. Singh et al. [44] reviewed active packaging techniques (e.g., antioxidant releaser, ethylene absorber, oxygen scavenger, carbon dioxide emitters) which support “clean label” trends with a focus on driving the “clean label” market. 

Consumers are more health-conscious about functional or 100% natural foods (such as certified health foods, whole grains, vegetables, etc.) [45,46,47]. Health-conscious consumers are more willing to buy organic food [48,49,50]. Health awareness of consumers will affect their attitudes when consuming food, and it will also affect consumers’ purchase intentions for a product. This literature highlights that health-conscious and health-oriented consumers, as well as consumers with above-average nutritional knowledge, typically read food labels more [51,52,53]. Therefore, this study will add the aspect of “product knowledge” to the discussion.

Schiffman and Kanuk [54] defined purchase intention as the possibility that determines the strength of customers’ willingness to purchase the commodity—the higher the possibility, the stronger the purchase intention. According to Strazzieri [55], the level of involvement (high or low) with the product under consideration depends on consumers’ decision processes. Therefore, most studies discuss purchase intention and involvement. Product knowledge also consistently ranks among the most important influences regarding consumer purchase behavior [56]. Product knowledge has a positive impact on consumer purchases. This study considers that the “clean label” is a type of recent new product. The public’s familiarity with and purchase intention for the “clean label” are difficult to estimate.

As implied by the existing studies, there seems to be a lack of sufficient research identifying factors influencing consumers’ awareness of the “clean label” system, which leaves several knowledge gaps that should be filled through further in-depth research. Therefore, the novel approach of this study is to use the three consumer aspects of product knowledge, involvement, and purchase intention to investigate consumers’ attitudes toward and opinion of the “clean label”, which was mentioned in—but was not tested by—previous studies, thereby filling this gap in the literature. Based on the results, recommendations are made for the food industry, product evaluation units, marketing strategies, or business management that are expected to bring a safer and healthier dietary environment to consumers.

In the rest of the paper, Section 2 will cover the literature on product knowledge, involvement, and purchase intention, and present hypotheses highlighting the relationships among them, as well as explaining the research methodology, which encompasses data collection, construction, and measurement. Further, Section 3 presents the data analysis process, which makes use of structural equation modeling (SEM). Next, Section 4 discusses the research and the managerial implications flowing from the findings. Finally, the limitations and suggestions for future research are expressed in the Conclusion.

## 2. Materials and Methods

### 2.1. Research Framework

**Figure 1:**
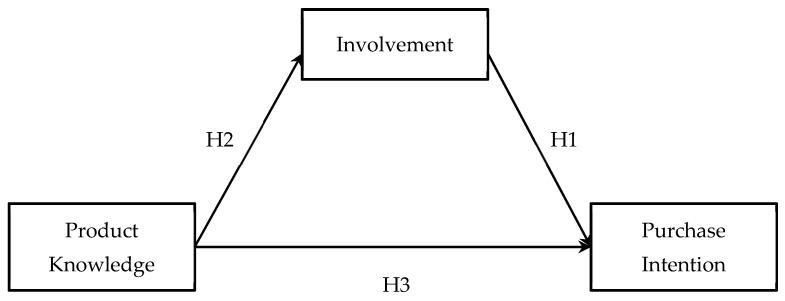
Research framework.

In this study, consumers’ purchase intention for the use of the “clean label” and its influencing factors are discussed, using product knowledge and involvement as independent variables. A conceptual model of this research is shown in Figure 1.

### 2.2. Research Hypotheses

#### 2.2.1. Purchase Intention

Schiffman and Kanuk [54] provided a definition of purchase intention as the possibility that determines the strength of customers’ willingness to purchase a commodity; a stronger possibility indicates a stronger purchase intention. Marketers have long argued that purchase intention can be used to accurately forecast purchase behavior [57,58,59].

According to Lee et al. [60], product attributes including product information, quality, and prices have positive effects on the purchase intention. Moreover, purchase intention is viewed as an antecedent to the actual purchase behavior [61]. The purchase intention is defined as the likelihood that a consumer will purchase a given product. A stronger purchase intention indicates an increase in purchase probability [54,62,63,64]. Thus, willingness to buy, and, henceforth, purchase intention, represents the probability that a consumer will purchase a product [65]. In other words, consumers with more positive information about a product or service are more intent on making a purchase, and the likelihood of a purchase increases substantially.

According to scholars, this study considers that purchase intention is affected by many factors, particularly for products that consumers are not familiar with or new products. Consumers’ purchase intention is more influenced by received knowledge messages and high or low levels of involvement, indicating that consumers’ purchase intention is related to product knowledge and involvement. 

#### 2.2.2. Involvement

Consumer product involvement is defined as: “consumers’ level of concern and attention for a product” [66]. Alternatively, we adopt Zaichkowsky’s [67] definition of involvement as: “A person’s perceived relevance of the object based on inherent needs, values, and interests”. According to Rahman [68], consumers are involved in a product when it has a significant substantial value for them or fundamental importance in their lives. 

Beharrell and Denison [69] noted that involvement influences the formation of beliefs, attitudes, and behavioral outcomes, such as the frequency of product usage, via considerable cognitive effort. For example, Celsi and Olson [70] demonstrated that “felt” involvement in a specific situation is a major driver of the attention process. In turn, this increased attention might lead to a deeper understanding of the sales dynamics that are peculiar to that particular product category. According to Strazzieri [55], the level of involvement (high vs. low) with the product under consideration depends on the consumer decision process. Hunjra et al. [71] stated that product knowledge has a positive association with product involvement, and its value is significant. Product knowledge also shows a significant association with purchase intention: the greater the consumer involvement, the more intensive is the use of information about these products [72]. Therefore, the more interested the participants, the higher their involvement. This study seeks to understand the degree of consumer cognition and attitude by consumer involvement in “clean label” products.

In summary, this study finds that the degree of involvement has an impact on purchase intention, and Hypothesis H2 is proposed.

**Hypothesis** **(H1).** *Product involvement positively affects consumer purchase intentions.*

#### 2.2.3. Product Knowledge

Such knowledge, according to Lin and Zhen [73], directly depends on consumers’ awareness or understanding of the given product, or on their confidence in the product. Furthermore, Coulter et al. [74] pointed out that their amount of product knowledge affects consumers’ choices of products and brands.

The consumers who have high product knowledge rely on intrinsic product characteristics to judge product quality and function. In contrast, the consumers with low product knowledge tend to evaluate a product based on its extrinsic cues, such as the price and brand [75]. Ghali-Zinoubi and Toukabri [76] considered product involvement as a vital construct of the study of consumer behavior and defined it as a “consumer’s enduring perceptions of the importance of the product category, derived from his innate desires, values, and interests”.

When engaged in the process of purchasing a given product, the knowledge that consumers have about the product affects their search behavior, their processing of relevant information, their decision-making, and, finally, their purchase intention [77,78]. Hence, product knowledge has a positive association with product involvement, and its value is significant [71]. Therefore, product knowledge consistently ranks among the most important influences regarding consumer purchase behavior (Burton et al.,2009).

As a result, this study argues that, if customers have more product knowledge, they have greater capabilities to distinguish the product’s quality. Additionally, these capabilities can reduce uncertainty to improve the feedback and purchase intention of products.

Therefore, we propose the following hypotheses.

**Hypothesis** **(H2).** *Product knowledge positively affects consumers’ involvement.*

**Hypothesis** **(H3).** *Product knowledge positively affects consumers’ purchase intention.*

### 2.3. Questionnaire Design

The questionnaire is divided into four parts, with parts 1, 2, and 3 addressing product knowledge, involvement, and purchase intention. First, the questions related to product knowledge refer to the study by Hunjra et al. [71] and Chen and Deng [78]. Next, the questionnaire addresses involvement, referring to the studies by Rahman et al. [79] and Hunjra et al. [71]. The third section addresses consumers’ purchase intention, referring to the research by Lee et al. [60], De Angelis et al. [64], and Ghali-Zinoubi and Toukabri [76]. They are measured using a seven-point Likert attitude scale representing strongly disagree (1) to strongly agree (7). A higher score indicates a higher evaluation of the attribution that the customer’s rate. Part 4 represents the respondents’ personal information, such as their gender, age, education level, and average monthly income.

### 2.4. Sample Size and Composition

This study is based on the three dimensions of product knowledge, involvement, and purchase intention to discuss the cognitions and opinions of “clean label” products of respondents who purchased these products and the impact on their purchase intention. The research method of this study is a web survey, and the formal questionnaire was sent to 292 participants. After eliminating the invalid samples, we retained 265 valid questionnaires for the analysis. The valid response rate is 90.75%.

After the data screening, the sample size was 265, of whom 61.10% are females and 38.90% are males. The ages in the sample were 21–30 years (24.15%), followed by 51–60 years (21.50%), and 41–50 years (20.75%). Regarding education level, university was the majority (60%), followed by high school (26.04%). The monthly income of the sample was 20,001–30,000 TWD (29.80%), followed by 30,001–50,000 (26.80%).

### 2.5. Statistical Analysis

This study adopted structural equation modeling (SEM) to measure the causal relationships between the latent variables. SEM is an effective model, test, and improvement method that enables theoretical models to be tested. SEM can explain the causal relationships among the variables for hypotheses related to models based on statistical dependence. Consumer behavior is often affected by psychological variables that cannot be directly estimated (latent variables), requiring observation variables to be measured indirectly. 

SEM is divided into two parts: the measurement model and the structural model. The measurement model is the part that relates the measured variables to the latent variables, and factor analysis is used for the evaluation. The structural model is the part that relates the latent variables to one another, and a path analysis is used for the evaluation. The empirical results are shown in the next section.

## 3. Results and Discussion

### 3.1. Measurement Model: Reliability and Validity

The results of the reliability and validity analysis of each variable are shown in Table 1. Nunnally [80] believed that each variable has a high reliability value if the Cronbach’s α coefficient is greater than 0.7 and has a low reliability value if the Cronbach’s α coefficient is lower than 0.35, and thus should be rejected. As for the construct validity, if the factor loading of each variable is higher than 0.5, it means that the item possesses construct validity [81]. According to the reliability factor, the overall reliability of the questionnaires in this study is greater than 0.7, indicating that the questionnaire data have high reliability. The average variance extracted (AVE) and the construct reliability (CR) are also up to the standard values. Furthermore, the mean, standard deviation, and correlations among the constructs are presented in Table 1.

### 3.2. Structural Model: Goodness-of-Fit Statistics and Hypothesis Testing

The proposed model was evaluated by running the SEM with the maximum likelihood estimation method. The findings indicate that our proposed model has a satisfactory predictive ability in the outcome variables. H1, which states that product involvement positively affects consumers’ purchase intention, is established (β = 0.304, *p* < 0.001). These results show that, after a consumer carefully assesses the difference between “clean label” and non-”clean label” products, the consumer’s purchase intention of “clean label” products increases. In response to this statement, Lee et al. [60] found that product attributes including product information, quality, and prices have positive effects on purchase intention. Hunjra et al. [71] stated that product involvement has a significant positive association with purchase intention. Most consumers take the initiative to positively search for product information and carefully compare and think about the differences between different brands when faced with high-involvement products. They expect to make the best purchase decisions and reduce the risk of mispurchases, which also confirms Hypothesis H1 of this study.

The results show that H2, which states that product knowledge positively affects consumers’ involvement, was first evaluated, and the results (β = 0.593, *p* < 0.001) support this hypothesis. According to the literature review, the degree of consumers’ product knowledge plays a very important role when they receive and process information. A study on dietary supplements showed that higher consumer involvement results in more intensive use of information about these products [72]. Moreover, product knowledge has a positive association with product involvement, and its value is significant [71]. This study extends these findings to “clean label” products and deduces the impact of product knowledge on the degree of involvement. According to the results of the questionnaire, although consumers have not bought or consumed “clean label” products, if consumers feel that the “clean label” is important or necessary, they will take the initiative to understand the function and related products with the “clean label”. This finding also confirms Hypothesis H2 of this study.

Last, Hypothesis H3 is examined, which states that product knowledge positively affects consumers’ purchase intention. The results (β = 0.317, *p* < 0.001) indicate that H3 is established. Coulter et al. [74] pointed out that the amount of product knowledge affects consumers’ choice of product and brand. Burton et al. [56] considered that product knowledge consistently ranks among the most important influences regarding consumer purchase behavior. Hunjra et al. [71] stated that product knowledge also has a significant association with purchase intention. This study also synthesizes various studies to conclude that “when consumers have more positive information about products or services, they may have the intent to purchase and the possibility of engaging in buying behavior significantly increases.” This finding confirms Hypothesis H3 of this study.

Most prior studies explored organic food labels and the impact of the involvement of organic vegetables and green diets on the purchase intention. Therefore, this study extends these prior findings to explore the effects of “clean label” products and the relevance of product knowledge, involvement, and purchase intention. The results also confirm that product knowledge and involvement have a significant positive impact on the purchase intention. Based on these findings, hypotheses H1, H2, and H3 of this study are established. A summary of the verification of the hypotheses made in this study is shown in Table 2.

**Figure 2:**
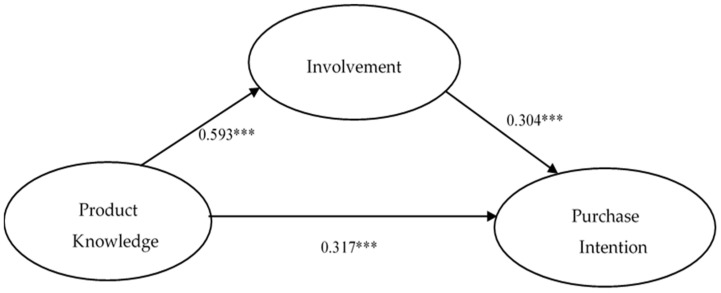
Results of structural equation modeling. *** *p* < 0.001; GFI = 0.907; TLI = 0.913; IFI = 0.943; RMSEA = 0.052.

The analysis began with a confirmatory factor analysis (CFA) using AMOS 21.0. The measurement model contains five latent constructs (Figure 2). After an initial CFA analysis, the revised model exhibited an appropriate level of model fit: x^2^/df = 2.328, the Goodness-of-Fit index(GFI) = 0.907, Tucker–Lewis Index (TLI) = 0.913, Incremental Fit Index (IFI) = 0.943, and the Root Mean Square Error of Approximation (RMSEA) = 0.052. All the other fit indices were above the recommended criteria. As a result, all the indices provided evidence of an acceptable measurement model. (Table 3).

## 4. Conclusions

Based on the results of the empirical analysis, this study suggests four main conclusions that can solve the problems and dilemmas that food industry and appraisal units may encounter.

### 4.1. Research Conclusions

1. Adapt to changes in dietary preferences and provide products that meet public expectations.

Food safety issues have continued for the last five years, and the public was no longer able to purchase delicate and delicious food. Instead, consumers began to choose natural and organic food. From the product and R&D perspective, the mainstream products in the market now emphasize health and nature. If food companies can change from these two aspects, they can not only meet the public’s needs but also increase customers’ confidence in products and their purchase intention by applying for the “clean label”.

2. Indicator selection for “clean label”

Even if products on the market have product labels, for customers to identify whether a product is natural and safe is still difficult and can only be determined by the brand or word-of-mouth. Therefore, the “clean label” is not only the recognition of natural products but also becomes one of the new bases for consumers to choose products. It can also be regarded as a way to transform food in a future direction. According to the references and the results of this study, an increase in customers’ product knowledge will enhance their involvement and improve purchase intention. Hence, we suggest making customers understand the significance of the “clean label” to strengthen marketing strategies. Therefore, spreading the concept of the “clean label” will help the public not only easily identify products but also reduce the uncertainty of new products. These results can increase the market of the food companies and enhance consumers’ purchase intentions.

3. Improve faith in the “clean label”

Since the food industries in Taiwan have experienced many food safety problems, the public has maintained a doubtful attitude toward labels, such as TQF. Thus, the “clean label” belongs to emerging food labeling. The data show that, although the public does not understand the “clean label”, they are willing to buy products with this mark after realizing its meaning. In 2015, the Institute of Food Technologists stated that the “clean label” would be a trend and was the only method to adopt. Currently, approximately 6–10% of foods throughout the world are being replaced by “clean label” products, and this percentage is estimated to continue to increase in the future. Compared with the data from this survey related to achieving the goal of promoting the “clean label”, if the appraisal unit could take the following actions, such as giving this mark greater conformability, improving relevant norms and standards, and strengthening people’s awareness and education, the influence and credibility of this mark will be significantly improved.

4. Popularization of “clean label”

Cooperating with Family Mart and increasing the cooperation with other marketing channels, such as Carrefour Mart, or other convenience stores that have developed their own brands actively in recent years, is suggested. Given the public’s sense of trust of well-known brands, a series of “clean label” products can be created to increase publicity and purchase intention. On top of that, people clearly need to recognize the idea of the “clean label” concept.

### 4.2. Research Limitations and Future Research Directions

After the data is collected and organized, there are still deficiencies in the research process. The data analysis and assumptions should be rigorous in order to obtain more accurate research results. Therefore, for the areas that should be strengthened in this research, the regulations are supplemented here for future research direction.

1. An important limitation of this study is the perception of the label information. Although the definition of “clean label” products was been clearly explained and illustrated with pictures at the beginning of the questionnaire of this study, this topic is new, and not every respondent shares the same understanding, which may make some respondents have biases when answering the questions.
2. To improve the accuracy of the research results and obtain complete information, subsequent researchers can use this study as a basis and collect data from various other market segments (e.g., different regions and cities) to increase the coverage of the sample. The extrapolation and reference value of the research results can be enhanced, which will be helpful for food industry managers to formulate marketing strategies for target markets and improve the research on the factors influencing the behavioral intention regarding “clean label” products. In addition, future studies could perform random sampling collection and use larger sample sizes. Moreover, as past studies have noted that peer communication through social media can significantly influence purchase intention [82], social media promotion should be used to explore the factors influencing consumer acceptance of “clean label” products in the future.
3. Consumers’ environmental concern is an inevitable trend in the pursuit of Earth’s sustainable development. In contrast, producers can also be research subjects to discuss the effects of food safety, health, and environmental friendliness on business development, and whether they will integrate the concept of “clean label” products into practices when developing a production plan, as they want to shoulder social responsibility to gain consumers’ attention, thereby improving their company’s image.
4. This study incorporates variables, such as product knowledge and involvement, to discuss Taiwanese people’s thoughts on purchasing “clean label” products and the factors influencing their purchase decisions. Future studies can incorporate variables, such as product trust, perceived risk, and perceived benefits, to make the overall research more complete.

## Figures and Tables

**Table 1:** Results for factor loading, reliability, and validity.

Item	Factor Loading	Cronbach’s α	AVE	C.R.
Product Knowledge		0.873	0.646	0.892
1. I know the function and purpose of the “clean label”.	0.803			
2. I will learn voluntarily about the product knowledge of the “clean label”.	0.815			
3. I can provide knowledge about the “clean label” to other people.	0.778			
4. I have had the experience of purchasing products with the “clean label”.	0.819			
5. I have had the experience of eating products with the “clean label”.	0.825			
Involvement		0.921	0.673	0.863
1. I am interested in products with the “clean label”.	0.832			
2. I know how many different types of “clean label” are available.	0.783			
3. I think products with the “clean label” can improve food safety.	0.846			
4. Products with the “clean label” are important for me.	0.854			
5. I need products with the “clean label”.	0.779			
6. I think the “clean label” system can be more complete.	0.791			
Purchase Intentions		0.916	0.682	0.905
1. The possibility of me buying a “clean label” product is high.	0.862			
2. Purchasing products with the “clean label” is my first choice.	0.762			
3. I will buy products with the “clean label”.	0.848			
4. I will recommend that people buy products with the “clean label”.	0.830			
5. The opinions of well-known professionals, such as doctors and dietitians, have influenced me to purchase products with the “clean label”.	0.782			

**Table 2:** Summary of hypothesis verification.

Hypothesis	Content	Verification
H1	Products’ involvement positively affects consumers’ purchase intention.	Supported
H2	Product knowledge positively affects consumers’ involvement.	Supported
H3	Product knowledge positively affects consumers’ purchase intentions.	Supported

**Table 3:** Summary of Goodness-of-Fit Indices for the Structural Models.

Model	*x*^2^/df	GFI	TLI	RMSEA	IFI
Structural model	2.328	0.907	0.913	0.052	0.943
Recommended value	≤3.00	≥0.90	≥0.90	<0.08	≥0.90

## Data Availability

The data that support the findings of this study are available from the corresponding author, H.-S.C., upon reasonable request.
